# The Role of Inflammation and Immune Activation on Circulating Endothelial Progenitor Cells in Chronic HIV Infection

**DOI:** 10.3389/fimmu.2021.663412

**Published:** 2021-05-17

**Authors:** Ziang Zhu, Tong Li, Jinya Chen, Jai Kumar, Princy Kumar, Jing Qin, Colleen Hadigan, Irini Sereti, Jason V. Baker, Marta Catalfamo

**Affiliations:** ^1^ Department of Microbiology and Immunology. Georgetown University School of Medicine, Washington, DC, United States; ^2^ Division of Infectious Diseases and Tropical Medicine, Georgetown University School of Medicine, Washington, DC, United States; ^3^ Biostatistics Research Branch, Division of Clinical Research (DCR), National Institute of Allergy and Infectious Diseases (NIAID), National Institutes of Health (NIH), Bethesda, MD, United States; ^4^ Laboratory of Immunoregulation, National Institute of Allergy and Infectious Diseases, National Institutes of Health, Bethesda, MD, United States; ^5^ Hennepin Healthcare Research Institute, University of Minnesota, Minneapolis, MN, United States

**Keywords:** endothelial progenitor cells, HIV infection, endothelial inflammation, T cell activation, endothelial repair

## Abstract

Endothelial inflammation and damage are the main drivers of cardiovascular risk/disease. Endothelial repair is mediated in part by recruitment of bone marrow endothelial progenitor/endothelial colony forming cells (EPC/ECFC). People with HIV (PWH) have increased cardiovascular risk and the impact of infection in endothelial repair is not well defined. The low frequencies and challenges to *in vitro* isolation and differentiation of EPC/ECFC from PBMCs had made it difficult to study their role in this context. We hypothesized that HIV driven inflammation induces phenotypic changes that reflects the impact of infection. To test this hypothesis, we evaluated expression of markers of trafficking, endothelial differentiation, and angiogenesis, and study their association with biomarkers of inflammation in a cohort of PWH. In addition, we investigated the relationship of circulating endothelial progenitors and angiogenic T cells, a T cell subset with angiogenic function. Using a flow cytometry approach, we identified two subsets of circulating progenitors LIN4^-^CD45^-^CD34^+^ and LIN4^-^CD45^dim^CD34^+^ in PWH. We found that the phenotype but not frequencies were associated with biomarkers of inflammation. In addition, the percentage of LIN4^-^CD45^dim^CD34^+^ was associated with serum levels of lipids. This data may provide a new tool to better address the impact of HIV infection in endothelial inflammation and repair.

## Introduction

In people with HIV (PWH), immune activation and inflammation is associated with cardiovascular risk independently of the traditional risk factors or duration of antiretroviral treatment and CD4 counts (1–12). PWH has twice the risk of cardiovascular disease compared to the general population and the standard cardiovascular risk prediction scores underestimate cardiovascular risk in these patients (1, 13).

Endothelial inflammation and injury play a critical role in the pathogenesis of cardiovascular risk/disease. The homeostasis, maintenance and repair of endothelial cells is mediated in part by bone marrow-derived endothelial cell progenitors also called endothelial colony forming cells (EPC/ECFC) (14–16). The low frequency, absence of specific markers, and the overlap with hematopoietic progenitor markers creates challenges for study this population in circulation (14, 17–20). In *in vitro* cultures of PBMC, two subsets of progenitor cells have been isolated, the “putative endothelial progenitor cells” called EPC/ECFC that can undergo clonal expansion, differentiate into endothelial cells, and promote neo-vasculogenesis (14, 21). The other subset of progenitor cells is the “angiogenic or colony forming unit endothelial cells” (CFU-EC). These cells have hematopoietic origin, and while they cannot differentiate into endothelial cells, they promote vasculogenesis by the secretion of critical growth factors (17, 22). In addition, recent evidence had shown that a subset of T cells can promote angiogenesis by secreting growth factors that drives proliferation and differentiation of endothelial progenitor cells. Because of these properties these T cells are called angiogenic T cells (T_angs_) (22, 23).

EPC/ECFCs have been studied as potential biomarker of disease and their levels in circulation have been associated with cardiovascular risk and events (24, 25). In contrast in PWH, studies of the frequencies of circulating EPC/ECFC have shown contradictory results and their role in HIV associated cardiovascular risk is not well defined (26–32). The challenges to isolate and culture *in vitro* endothelial progenitor cells from PWH suggest that in this setting some functional properties of these cells are compromised (29). Accordantly, one study reported that factors present in the serum from PWH alters the functional properties of EPC/ECFC isolated from cord blood of healthy volunteers. These observations propose that systemic inflammation in the setting of HIV infection can influence the functionality with no changes in their frequency (33).

In this study, we hypothesize that systemic inflammation and immune activation induces changes in their phenotype that reflects the impact of HIV infection. To address this question, we developed a high parameter flow cytometry approach to study the relationships between the phenotype of endothelial progenitors and serum levels of biomarkers of inflammation, and angiogenic T cells.

## Material and Methods

### Participants

Participants (PWH, n= 36) were recruited at the HIV Clinic in Minneapolis, Minnesota (Hennepin Healthcare) under an institutional review board approved protocol (**Table 1**). Biomarkers and clinical chemistry are described in **Supplementary Material and Methods**. Patients and healthy controls were consented and studied in NIAID/CCMD intramural program IRB approved HIV clinical research studies (**Supplementary Table S1**). Healthy volunteers were obtained through the NIH Blood Bank and were recruited at the MedStar University Hospital (n = 6). Georgetown University. All participants signed informed consent.

**Table 1:** Patient Characteristics.

	Patients (n = 36)
**Age, yr,** median (IQR)	52 (45-57)
**Gender n (%)**	
male	31 (86.1)
female	5 (13.9)
**Race/Ethnicity n (%)**	
White	24 (66.7)
African American	2 (5.6)
Hispanic	8 (22.2)
Other	2 (5.6)
**Clinical Characteristics**	
Smoker, n (%)	12 (33.3)
Diabetes, n (%)	5 (13.8)
Hypertension diagnosis, n (%)	11 (30.6)
Lipid-lowering therapy (%)	12 (33.3)
Statins n (%)	11 (30.6)
Aspirin n (%)	10 (27.8)
BMI Kg/m^2^, median (IQR)	27.03 (24.46, 32.62)
SBP mmHg, median (IQR)	130 (121, 140.5)
DBP mmHg, median (IQR)	79 (74, 83)
FRS 10 yr %, median (IQR)	11.2 (9, 18.4)
**HIV infection, median years, (IQR)**	14 (10, 20)
**T cell counts, median (IQR)**	
CD4 counts (cells/μL)	662 (503, 856)
CD8 counts (cells/μL)	557.5 (436.5-913.8)
CD4 nadir (cells/μL)	331.5 (91.25, 404.5)
CD4/CD8 ratio	1.03 (0.734, 1.58)
**ART, n (%)**	
Tenofovir	24 (64)
Abacavir	11 (30.6)
NNRTI	11 (30.6)
PI	13 (36.1)
INSTI	17 (47.2)
**Clinical Laboratory, median (IQR)**	
Total Cholesterol mg/dL	184.5 (145,.5, 216.3)
LDL mg/dL	108 (79, 129,3)
HDL mg/dL	46 (34, 64.75)
Triglycerides mg/dL	123.5 (85.75, 177.5)
**Biomarkers, median (IQR)**	
IL-8 (pg/mL)	3.482 (2.718, 4.843)
hsIL-6 (pg/mL)	1.806 (1.28, 2.527)
IL-6R (ng/mL)	38.46 (31.69, 46.410)
TNFα (pg/mL)	2.491 (1.896, 3.129)
TNFRI (ng/mL)	2.784 (2.297, 3.1740)
hsCRP mg/mL	1.223 (0.6645, 2.566)
sCD163 (mg/L)	0.1769 (0.1414, 0.265)
sCD14 (mg/L)	1.705 (1.561, 1.88)
D-dimer mg/L	0.3512 (0.2362, 0.4547)
TFPI (ng/mL)	31.2 (28.7, 37.13)
sICAM1 (µg/mL)	332.2 (259.6, 407.7)
sVCAM1 (µg/mL)	407.7 (360.1, 552.1)

SBP, Systolic Blood Pressure; DBP, Diastolic Blood Pressure, ART, antiretrovirals, FRS, Framingham Risk Score; DM, Diabetes Mellitus, BMI, Body mass index, LDL, Low-Density Lipoprotein cholesterol; HDL, High-Density Lipoprotein cholesterol; IL-8, interleukin 8; IL-6, interleukin 6; IL-6R, interleukin 6 Receptor; TNF, Tumor Necrosis Factor; TNFRI, Tumor Necrosis Factor Receptor I, hsCRP, high sensitivity C-Reactive Protein; sCD163, soluble CD163; sCD14, soluble CD14; TFPI ,Tissue Factor Pathway Inhibitor; sICAM1, soluble intercellular adhesion molecule1; sVMAC1, soluble vascular adhesion molecule1.

### Flow Cytometry

Frozen PBMCs from PWH and healthy controls were thawed and rested overnight. Cells were stained with LIVE/DEAD (Invitrogen, CA). Cells were incubated with 1μg/mL human IgG (Sigma, MO) to block Fc receptors followed by a cocktail of mAbs (**Supplementary Materials and Methods**, **Supplementary Table S2**). Samples were acquired with a BD Symphony and analyzed using Flowjo software.

### Statistical Analysis

Associations between circulating progenitor cell subsets and serum levels of biomarkers (**Supplementary Table S3–S5**) were performed using Pearson Correlation Coefficient and two sample t-test. Because of the multiple comparison *p*-value <0.01 was considered significant.

## Results

### Two Circulating Subsets LIN4^-^CD45^-^CD34^+^ and LIN4^-^CD45^dim^CD34^+^ of Progenitor Cells Are Detected in PBMCs From PWH

The EPC/ECFC have been defined by the expression of CD34^+^KDR^+^ (CD309), however, these markers are expressed by other cells (14, 20, 34). From *in vitro* cultures of PBMCs, two progenitors have been identified, the EPC/ECFC have been defined as CD45 negative whereas, the angiogenic or colony forming unit endothelial cells (CFU-EC), which are hematopoietic origin and express CD45 (14, 15, 18, 19, 22).

To overcome the challenges to isolation and culture EPC/ECFC cells from PBMCs from PWH and determine the impact of infection, we developed a flow cytometric panel that allow us their detection in frozen PBMCs, and to study their phenotype. Because of the low frequencies, the overlap of surface makers with different cell types in blood that can lead to false positive events we used a cocktail of mAbs (LIN4) to exclude all the lineage cells from hematopoietic origin (**Supplementary Material and Methods Table S2** and **Supplementary Figure S1**). Using this approach, we identified two subsets of cells expressing CD34^+^ similar to that described in the cultures of PBMCs (14, 15, 18, 19, 22). One population was CD45^-^ (LIN4^-^CD45^-^CD34^+^) and one expressed CD45^+^ gate (expressing CD45^dim^) and through the manuscript we call this population as LIN4^-^CD45^dim^CD34^+^ (**Supplementary Figure S1**).

Having validated this flow cytometry panel in frozen PBMCs, we next analyzed the frequency of circulating progenitor in a cohort of 36 PWH with a median time from the diagnosis of HIV infection 14 (10, 20) years (**Table 1**). Participants had a median age 52 (45-57) years, 86% were male sex at birth, 67% were white, 33% smokers and median BMI was 27 kg/m2 and a moderate Framingham Risk Score (FRS) median 11.2. All PWH were receiving cART for more than a 1 year with HIV-RNA levels <200 copies/mL and have a median CD4 T cell count of 662 cells/μL (**Table 1**). The clinical and laboratory characteristics, biomarkers of inflammation, coagulation and vascular inflammation are described in **Table 1**.

**Figure 1:**
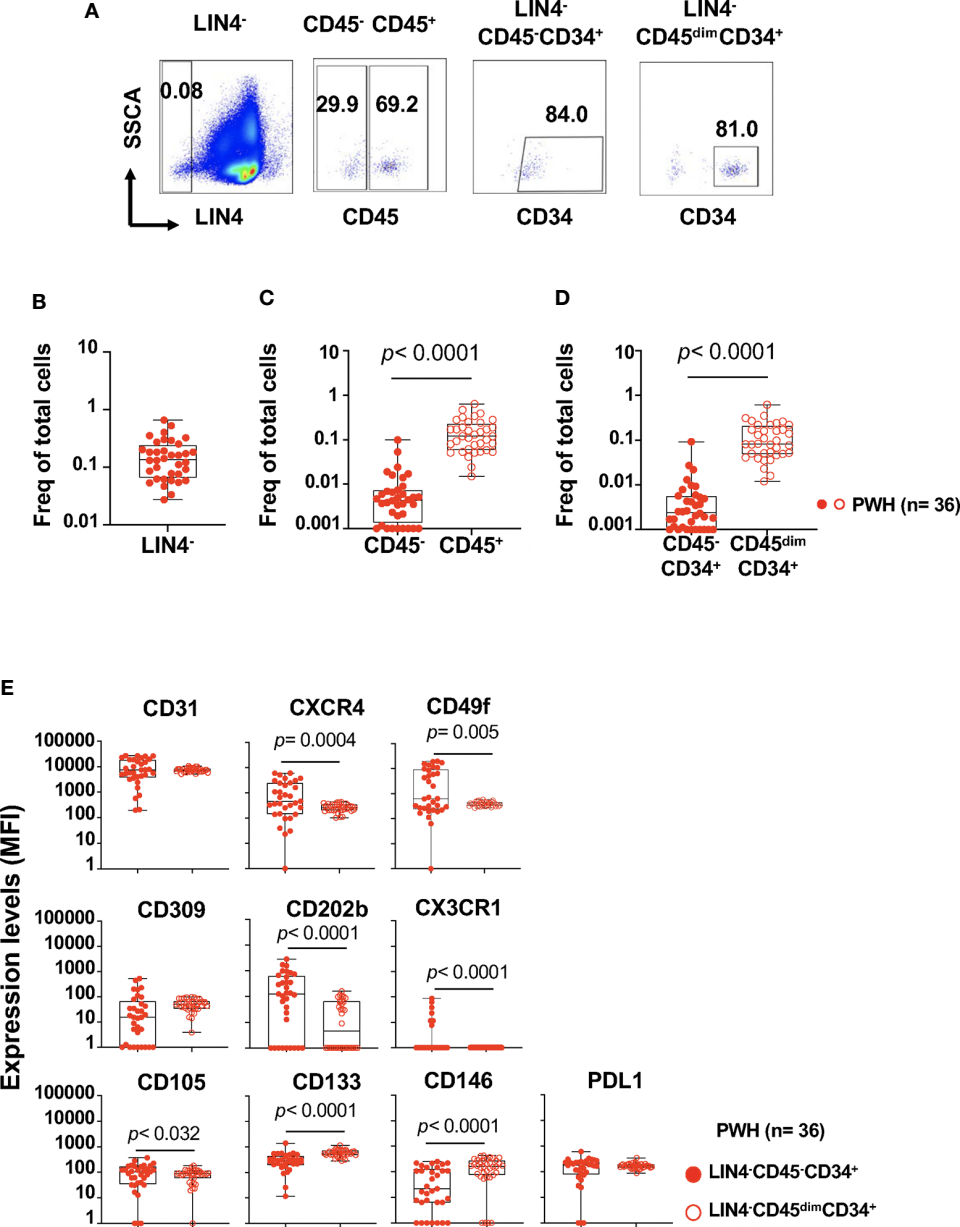
Detection and phenotype of two circulating cell progenitors LIN4^-^CD45^-^CD34^+^ and LIN4^-^CD45^dim^CD34^+^ in PBMCs from PWH. PBMCs from PWH (n= 36) were thawed and rested overnight. PBMCs were stained with LIVE/DEAD followed by a cocktail of mAbs: LIN4, CD45, CD3, CD8, CD34, CD31,CD105, CD49f, CD133, CD146, PDL-1, CD14, CD309 and CD202b (**Supplementary Table S2**). Full minus one (FMO) was used control. **(A)** Representative dot plots of the gating strategy. Frequency of: **(B)** LIN4^-^, **(C)** LIN4^-^CD45^-^ and LIN4^-^CD45^+^, **(D)** LIN4^-^CD45^-^CD34^+^ and LIN4^-^CD45^dim^CD34^+^ cells are represented as the frequency of total live cells. **(E)** Expression of the markers CD31, CXCR4, CD49f, CD309, CD202b, CX3CR1, CD105, CD133, CD146 and PD-L1 in the LIN4^-^CD45^-^CD34^+^ (closed red circle) and LIN4^-^CD45^dim^CD34^+^ (open red circle) cells are shown as median fluorescence intensity (MFI). Whiskers represent median and IQR. Comparison between subsets was performed using non-parametric Wilcoxon test. *P* value < 0.05 was considered significant.

The proportion of LIN4^-^ cells (expressed as the frequency of total live cells) in PBMCs from PWH was % 0.140 (IQR: 0.065 to 0.240), as depicted in **Figure 1B** (**Figure 1A** gating strategy). Lower frequencies of the LIN4^-^CD45^-^ compared to the LIN4^-^CD45^+^ subset were observed (**Figure 1C**).

The frequency of the LIN4^-^CD45^-^CD34^+^ progenitor cells were lower than LIN4^-^CD45^dim^CD34^+^ subset, *p*< 0.0001 (**Figure 1D**). In addition, stable frequencies of these subsets in PBMCs were measured in two time points of an interval of six months apart (**Supplementary Figure S2**).

These results showed that two circulating CD45^-^CD34^+^ and CD45^dim^CD34^+^ progenitor cells can be detected during chronic HIV infection, and steady frequencies were observed during approximately 6 months interval of stable virologic control.

### Circulating LIN4^-^CD45^dim^CD34^+^ Progenitor Cells Are Associated With Levels of Lipids During HIV Infection

To evaluate the impact of HIV infection on LIN4^-^CD45^-^CD34^+^ and LIN4^-^CD45^dim^CD34^+^ subsets, we next studied the association between the proportion of circulating cell progenitors and biomarkers of inflammation and coagulation, lipids profile, and cardiovascular risk (**Supplementary Tables S3, S4**). No associations were observed between the frequency of LIN4^-^CD45^-^CD34^+^ and LIN4^-^CD45^dim^CD34^+^ subsets and the plasma levels of biomarkers of inflammation (**Supplementary Table S3**).

In contrast, the frequency of circulating LIN4^-^CD45^dim^CD34^+^ subset showed a statistically significant although weak associations with the levels of total cholesterol (R= 0.533, *p*= 0.001), LDL cholesterol (R= 0.647, *p*<0.001), and triglycerides (R= 0.466, *p*= 0.004), **Supplementary Table S4**. To better understand these relationships, we performed a multivariate analysis using DM (diabetes), total cholesterol, LDL cholesterol and triglycerides as variables. We found that the frequency of LIN4^-^CD45^dim^CD34^+^ was associated with LDL cholesterol, R^2^ = 0.44, *p*< 0.01 (estimated coefficient 0.00277, 95% CI 0.00085, 0.00469) and triglycerides, R^2^ = 0.29, *p*< 0.01 (estimated coefficient 0.00065, 95% CI 0.00023, 0.00107).

These data suggest that the levels of biomarkers of systemic and endothelial inflammation may not influence the frequencies of cell progenitors in circulation in this study group of PWH. In addition, the results highlight a potential link between serum lipids levels and the frequencies of LIN4^-^CD45^dim^CD34^+^ but not LIN4^-^CD45^-^CD34^+^ progenitor cells.

### Expression of Endothelial Markers by Circulating Endothelial Progenitors Are Associated With Biomarkers of Inflammation

One limitation to study the *in vivo* functional properties of endothelial progenitors in PWH is the challenge to *in vitro* culture and differentiate them from PBMCs (30, 33). In addition, the *in vitro* culture may select clones that can proliferate and differentiate in endothelial progenitor cells and may not represent the impact of the infection in the overall population of cell progenitors. To overcome these challenges, we hypothesized that the inflammatory environment associated with HIV infection is reflected in their phenotype.

We developed a multidimensional flow cytometry approach to study the relationships between the expression levels of markers associated with progenitor cells (CD34, CD133, CD49f), endothelial cell markers (CD31, CD105, CD309, CD202b, CD146, PD-L1), and homing receptors (CXCR4, CX3CR1); and serum levels of biomarkers of inflammation (**Supplementary Table S2** and **Figure 1E**).

We found that both LIN4^-^CD45^-^CD34^+^ and LIN4^-^CD45^dim^CD34^+^ expressed the homing receptors CD31, CXCR4 and CD49f (the laminin receptor) involved in trafficking and homing of progenitor cells. In addition, LIN4^-^CD45^-^CD34^+^ showed significant higher expression levels of CXCR4, CD49f, CD202b, CD105 and CX3CR1 than the LIN4^-^CD45^dim^CD34^+^ subset (**Figure 1E**). In contrast, LIN4^-^CD45^dim^CD34^+^ subset expressed higher levels of the CD133 and CD146 surface markers. Similar expression levels between the subsets were observed for CD31, CD309 and PD-L1 (**Figure 1E**). The analysis of the frequency of cell subsets expressing these markers showed similar results with the exception of CD202b expression. The frequency of CD202b^+^ LIN4^-^CD45^dim^CD34^+^ cells expression was higher than LIN4^-^CD45^-^CD34^+^ (**Supplementary Figure S3B**).

We next determine the potential influence of systemic inflammation in the phenotype of the progenitors (**Supplementary Table S5**). We found a positive association, although weak, between the serum levels of CRP and the expression of endothelial markers CD309 (R= 0.582, *p*= 0.002) and PD-L1 (R= 0.507, *p*= 0.004) in LIN4^-^CD45^-^CD34^+^ subset. In addition, the serum levels of IL-6 were associated with expression of CD105 (R= 0.465, *p*= 0.009).

In contrast in the LIN4^-^CD45^dim^CD34^+^ subset, the expression of PD-L1 was positively associated with the serum levels of IL-6 (R= 0.459, *p*= 0.005), TNFRI (R= 0.454, *p*= 0.005), **Supplementary Table S5**. No associations were observed with endothelial inflammation biomarkers including sICAM, and sVCAM (data not shown).

Altogether these data suggest that in the context of HIV infection, biomarkers of inflammation have a potential effect on the expression of markers associated with endothelial differentiation in both LIN4^-^CD45^-^CD34^+^ and LIN4^-^CD45^dim^CD34^+^ subsets.

**Figure 2:**
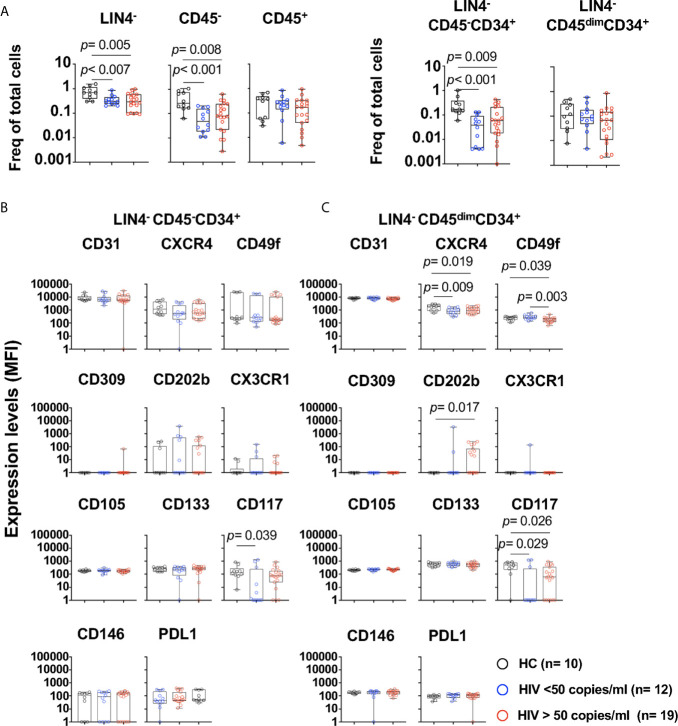
Phenotype of circulating cell progenitors LIN4^-^CD45^-^CD34^+^ and LIN4^-^CD45^dim^CD34^+^ in PWH. PBMCs from healthy control (HC, n=10), PWH with viral loads < 50 copies/ml (n= 12), and > 50 copies/ml (n= 19) were thawed and rested overnight. PBMCs were stained with LIVE/DEAD followed by a cocktail of mAbs: LIN4, CD45, CD34, CD31, CXCR4, CD105, CD49f, CD133, CD146, PDL-1, CD309, CD117 and CD202b (**Supplementary Table S2**). **(A)** Percentage of LIN4^-^, LIN4^-^CD45^-^ and LIN4^-^CD45^+^(Left panel), and percentage of LIN4^-^CD45^-^CD34^+^ and LIN4^-^CD45^dim^CD34^+^ (Right panel). Percentages are represented as frequency of total live cells. Expression of the markers in: **(B)** LIN4^-^CD45^-^CD34^+^, and **(C)** LIN4^-^CD45^dim^CD34^+^ cells. Expression of the markers as shown as median fluorescence intensity (MFI). Whiskers represent median and IQR. Comparison between groups was performed using non-parametric Mann-Whitney test. *P* value < 0.05 was considered significant.

We next evaluated whether HIV viral replication and its associated inflammatory environment has an effect in the phenotype of these subsets (**Supplementary Table S1** and **Figure 2**). PWH with viral loads > 50 copies/ml showed significant lower frequencies of LIN4^-^, LIN4^-^CD45^-^ and LIN4^-^CD45^-^CD34^+^ compared to those with suppressed viral replication by cART and healthy volunteers (**Figure 2A**). In contrast, no changes were observed on the LIN4^-^CD45^+^ and LIN4^-^CD45^dim^CD34^+^ (**Figure 2A**). The phenotype of the subset LIN4^-^CD45^-^CD34^+^ showed similar levels of expression except for the expression of CD117 (**Figure 2B** and **Supplementary Figure S4A**). In contrast, reduced expression levels of CXCR4, CD49f and CD117 was noted in the LIN4^-^CD45^dim^CD34^+^ subset (**Figure 2C** and **Supplementary Figure S4B**).

The frequencies of LIN4^-^CD45^-^CD34^+^ or the LIN4^-^CD45^dim^CD34^+^ subset were not associated with the viral load, CD4 and CD8 T cell counts or with the expression of HLADR^+^CD38^+^ in T cells. Moreover, weak associations, although significant were observed with the phenotype of the cell subsets. CD4 T cell counts showed association with expression of CD146 expression (R= 0.498, *p*= 0.005) in the LIN4^-^CD45^-^CD34^+^ subset. In addition, the percentage of CD4^+^HLADR^+^CD38^+^ was associated with expression of CD117 in this subset (R= 0.652, *p*< 0.001). In the LIN4^-^CD45^dim^CD34^+^, viral load showed a trend of a negative correlation with the expression levels of CD49f (R= -0.420, *p*= 0.022), and the frequency of CD4 and CD8 HLADR^+^CD38^+^ was negatively associated with the expression of CD31 (R= -0.465, *p*=0.014 and R=- 0.584, *p*= 0.001 respectively). These results suggest that HIV and its associated immune activation may have an impact in the phenotype of these circulating cell subsets.

### CX3CR1^+^ Angiogenic T Cells and Circulating Endothelial Progenitors in PWH

Angiogenic T cells (T_angs_) can promote vascular repair by secretion endothelial growth factors and are defined by expression of CXCR4^+^CD31^+^ (23). We next evaluated the potential relationship between angiogenic T cells and circulating progenitors in PWH (**Table 1**).

**Figure 3:**
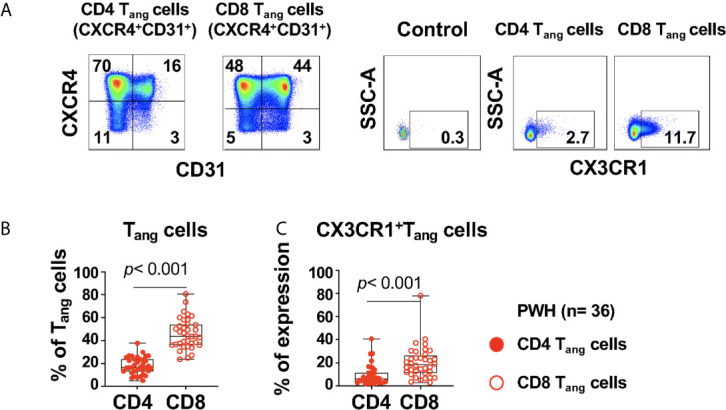
Expression of CX3CR1^+^CD4 and CD8 T_ang_ cells in PWH. PBMCs from PWH (n= 36) were thawed and rested overnight. PBMCS were stained with LIVE/DEAD followed by a cocktail of mAbs described in Table Flow Cytometry Panel (**Supplementary Table S2**). CD4 T cells were gated as CD3^+^CD8^-^ and CD8 T cells were gated as CD3^+^CD8^+^. **(A)** CD4 and CD8 angiogenic T cells (T_ang_) were identified based on surface expression of CXCR4^+^CD31^+^. Surface expression of CX3CR1 were analyzed in CD4 and CD8 T_ang_ cells and full minus one (FMO) was used control. **(B)** Percentage CD4 and CD8 T_ang_ cells. **(C)** Expression of CX3CR1 in CD4 and CD8 T_ang_ cells expressed as frequency of the parent population. Comparison between CD4 and CD8 T_ang_ cells was performed using nonparametric Wilcoxon test. *P* value < 0.05 was considered significant.

The study participants had a frequency of CD4 T_angs_ of 16.5% (IQR: 13.3 to 23.65), and CD8 T_angs_ 43.9% (IQR: 36.2 to 54.08), [**Figure 3B** (**Figure 3A** gating strategy)]. In addition, the frequency of CD4 and CD8 T_angs_ showed a trend of a negative correlation with the Framingham Risk Score (FRS) but did not rich statistical significance (R= -0.346, *p*= 0.045; and R= -0.347, *p*= 0.044 respectively, **Supplementary Table S6**).

CD4 T_ang_ cells were negatively associated with SBP (R= -0.510, *p*< 0.001) with a trend of association with DBP (R= -0.407, *p*= 0.014), and D-dimer levels (R= -0.407, *p*= 0.015). In contrast, CD8 T_ang_ cells showed a trend of negative association with SBP and DBP (R= -0.377, *p*= 0.023, R= -0.391, *p*= 0.018 respectively) but did not rich statistically significance (**Supplementary Table S6**).

CD4 and CD8 T_angs_ cells showed no correlation with the frequencies of circulating progenitors LIN4^-^CD45^-^CD34^+^ and LIN4^-^CD45^dim^CD34^+^ subsets (data not shown).

We next evaluated the expression of CX3CR1 in T_ang_ cells that has been reported as a marker of pro-inflammatory T cells and can mediate endothelial inflammation in the context of HIV infection (35). A higher proportion of CX3CR1^+^CD8 T_ang_ cells compared with their CD4 counterpart was observed in PWH (**Figures 3A, C**). In addition, we found that CX3CR1^+^ CD8 T_ang_ cells showed a trend of a positive correlation with circulating LIN4^-^CD45^-^CD34^+^ but did not rich statistical significance (R= 0.327, *p*= 0.052). These results suggest that in the setting of HIV infection, CD8 T_ang_ cells express CX3CR1 and have the potential to promote endothelial inflammation.

## Discussion

Endothelial inflammation is the underlying mechanism of cardiovascular risk/disease. Endothelial repair is mediated in part by recruitment of bone marrow derived progenitor cells, and the impact of HIV infection in this process is not well defined (30, 33, 36).

Several limitations to study the role of endothelial progenitors during HIV infection includes their low frequencies in circulation, the lack of specific markers and difficulties of *in vitro* culture from PBMCs. In an attempt to overcome these challenges and to better defined the *in vivo* impact of HIV infection, we investigated their phenotype and the relationships with biomarkers of inflammation.

We developed a high dimensional flow cytometry assay with a stringent exclusion of potential hematopoietic lineage contaminants to determine the frequencies and phenotype of circulating progenitor cells. One advantage of this approach is the distinction of two subsets of circulating progenitors (LIN4^-^CD45^-^CD34^+^ and LIN4^-^CD45^dim^CD34^+^) that are mostly not made in the previous reported studies of endothelial progenitors (36, 37). Our findings are in agreement with the observations from Echeveria et al. They reported circulating angiogenic cells defined as CD31^+^CXCR4^+^CD34^+^CD45^low^ cells, and a heterogenous population CD31^+^CXCR4^+^CD34^+^CD45^-^ containing circulating endothelial cells and endothelial progenitors in PWH (38). This study and our highlight the heterogeneity of circulating cell subsets associated with angiogenesis and vascular repair mechanisms.

Similar to previous reports, we found no associations between the frequencies of circulating progenitors and inflammatory biomarkers (30, 32). These inflammatory markers reflect endothelial damage and may not be involved in the mobilization and recruitment of cell progenitors (39). In addition, our study participants had a moderate FRS and although statistically significant, weak correlations were observed with the biomarkers studied. Therefore, future studies should evaluate cohorts with distinct cardiovascular risk to better define these relationships. We also evaluated the impact of viremia, and although we found reduced frequencies in circulation, no association between the viral load and the frequencies of these subsets were observed.

We observed an association between pro-atherogenic lipids including LDL and triglycerides, and the frequency of the LIN4^-^CD45^dim^CD34^+^ subset. This subset contains the “angiogenic” myeloid progenitors (CD45^dim^). Elevated lipids levels has been reported to be involved in the mobilization of hematopoietic stem and progenitor cells (40–42). Studies had shown that excess of circulating lipids drive endothelial inflammation, and particularly, LDL cholesterol is a modulator of recruitment of hematopoietic progenitors to the atherosclerotic plaques. These myeloid progenitors can differentiate into monocytes and macrophages and thereby contribute to the pathology of the vascular disease (41, 42). In addition, other studies shown that HDL cholesterol is a determinant of the number of endothelial progenitors in circulation (43).

In contrast to the frequencies, we observed that expression of markers of endothelial differentiation (CD309 and PD-L1) in the LIN4^-^CD45^-^CD34^+^ were associated with biomarkers of systemic inflammation including IL-6, CRP, whether these correlations reflect an ongoing differentiation process and altered functional property needs to be determined. Accordantly, a report showed that factors present in the serum of PWH but not in uninfected individuals alter the functional properties of normal donor cord blood derived endothelial progenitor cells (33). Particularly, they found that the plasma levels of CRP in untreated HIV infected patients was negatively correlated with functional properties of endothelial progenitors. The study did not report isolation of EPC/ECFC from PWH (33).

In addition, other factors can influence the functionality of these cells including the cART. The HIV protease inhibitor Ritonavir have been shown to have cytotoxic effects *in vitro* in human endothelial cells (44). More importantly, in a longitudinal study it was evaluated the impact of the commonly used protease inhibitors and non-nucleoside reverse transcriptase inhibitors on circulating endothelial cells, EPC/ECFC and angiogenic cells (38). In this study, the authors reported a greater recovery of circulating endothelial cells and EPC/ECFC in the group treated with the protease inhibitor darunavir suggesting an active endothelial repair process in the setting of cART (38).

In the present study, we also evaluated the potential relationship of endothelial cell progenitors, and subset of T cells with angiogenic properties (23). The study showed a trend in the relationship between LIN4^-^CD45^-^CD34^+^ and angiogenic CD8 T_ang_ cells expressing CX3CR1. CD8 T cells expressing CX3CR1 have been associated with the recruitment of pro-inflammatory T cells and endothelial inflammation in the setting of HIV infection (35, 45). The role of CX3CR1 in angiogenic T cell function is not well defined, in animal models, CX3CR1 expression is associated with enhanced atherosclerosis and renal impairment suggesting a pro-inflammatory potential of these cells (46). In addition, we found an association between CD4 T_ang_ cells and blood pressure. Reports had indicated that T cells are involved in regulating blood pressure and in the context of HIV infection, the T cell immune activation may alter these mechanisms (47, 48).

Altogether this study suggests a complex interplay between endothelial inflammation and repair and a potential role of T cell immune activation in the setting of HIV infection. The evaluation of the phenotype of endothelial progenitor cells provides a new tool to a better assessment of these interactions and their contribution in cardiovascular risk in PWH.

## Data Availability Statement

The original contributions presented in the study are included in the article/**Supplementary Material**. Further inquiries can be directed to the corresponding author.

## Ethics Statement

Individuals with HIV were recruited at the HIV Clinic in Minneapolis, Minnesota (Hennepin Healthcare) under an institutional review board approved protocol (#13-3657). Healthy volunteers’ samples were obtained at the MedStar University Hospital under an institutional review board approved protocol (CR00000926). Participants from NIAID/CCMD intramural program IRB approved (91-I-0140). The patients/participants provided their written informed consent to participate in this study.

## Author Contributions

MC designed the study. ZZ, JC, and TL performed the experiments. MC, ZZ, and TL analyzed and interpreted the data and wrote the manuscript. JK, PK, CH, IS, and JB were involved in recruitment of participants of the study and wrote the manuscript. JQ performed statistical analysis. All authors contributed to the article and approved the submitted version.

## Funding

Research reported in this publication was supported by the National Institute of Allergy and Infectious Diseases of the National Institutes of Health under award number NIH R01AI145549-02. MC is supported in part by Leidos Biomedical Research, Inc. has been funded in whole or in part with federal funds from the National Cancer Institute, NIH, under Contract HHSN261200800001E. The content of this publication does not necessarily reflect the views or policies of the Department of Health and Human Services, nor does mention of trade names, commercial products, or organizations imply endorsement by the U.S. Government. MC is also supported in part by the District of Columbia Center for AIDS Research, an NIH funded program (P30AI117970), which is supported by the following NIH Co-Funding and Participating Institutes and Centers: NIAID, NCI, NICHD, NHLBI, NIDA, NIMH, NIA, NIDDK, NIMHD, NIDCR, NINR, FIC and OAR. The content is solely the responsibility of the authors and does not necessarily represent the official views of the NIH.

## Disclaimer

The content of this publication does not necessarily reflect the views or policies of the Department of Health and Human Services, nor does mention of trade names, commercial products, or organizations imply endorsement by the U.S. Government.

## Conflict of Interest

The authors declare that the research was conducted in the absence of any commercial or financial relationships that could be construed as a potential conflict of interest.
