# Supplementary material for: The Role of Inflammation and Immune Activation on Circulating Endothelial Progenitor Cells in Chronic HIV Infection
Source: Front Immunol. 2021 May 17;12:663412. doi: 10.3389/fimmu.2021.663412 (PMC8165313; doi:10.3389/fimmu.2021.663412)
Supplement: Supplementary file 1 [file Presentation_1.pdf]

Supplementary Materials

Circulating endothelial progenitor cells in chronic HIV infection

Ziang Zhu[1*], Tong Li[1*], Jinya Chen[1], Jai Kumar[2], Princy Kumar[2], Jing Qin[3], Hadigan C[4], Sereti I[4], Baker JV[5] and Marta Catalfamo[1]

[*]These authors had contributed equally to this work

[1]Department of Microbiology and Immunology. Georgetown University School of Medicine. Washington DC, USA

[2]Division of Infectious Diseases and Travel Medicine, Georgetown University School of Medicine. Washington DC, USA

[3]Biostatistics Research Branch, DCR, NIAID, National Institutes of Health, Bethesda, MD, USA

[4]Laboratory of Immunoregulation, National Institute of Allergy and Infectious Diseases, National Institutes of Health, Bethesda, MD, USA

[5]Hennepin Healthcare Research Institute. University of Minnesota, Minneapolis, Minnesota, USA.

Supplementary Materials

Extended Material and Methods

Biomarkers

Biomarker were measured with ELISA and MesoScale multiplex platforms from cryopreserved plasma. High sensitivity interleukin-6 (IL-6; ELISA, R&D Systems), soluble IL-6 receptor (sIL-6R, MesoScale), high sensitivity C-reactive protein (CRP; MesoScale), tumor necrosis factor receptor-1 (TNFR1; Mesoscale), TNFα, and IL-8 (Pro-Inflammatory II Panel, MesoScale). Monocyte activation markers were measured by ELISA, sCD14 (R&D Systems) and sCD163 (Aviscera Bioscience). sICAM and sVCAM by ELISA (R&D Systems). Biomarkers of coagulation, tissue factor pathway inhibitor (TFPI; R&D Systems) and D-dimer immuno-turbidimetric assay (Sta-R analyzer, Liatest D-DI, Diagnostic Stago).

Table S1. Patient Characteristics

	HIV$^+$ > 50 copies/ml n= 18#	HIV$^+$ < 50 copies/ml n= 12
**Age, median (IQR)**	58 (59, 61)	58 (59, 61)
**Gender n (%)**		
Male	16 (82)	9 (75)
Female	2 (18)	2 (25)
**Race/Ethnicity n (%)**		
White	11 (61)	6 (50)
Black	6 (33)	3 (25)
Other	1 (6)	2 (25)
**ART, n (%)**		
Tenofovir	6 (33)	9 (75)
NNRTI	2 (11)	5 (42)
PI	6 (33)	6 (50)
**Therapy months**	< 2 months*	44 (21.5, 85.75)
**T cell counts, median (IQR)**		
CD4 counts (cells/μL)	352 (237.8, 493.5)	406.5 (272.5, 698.8
CD8 counts (cells/μL)	958 (678, 1469)	792.5 (498.8, 1095)
**VL (copies/ml)**	6945 (8313, 26826)	< 50
**% CD4 HLADR$^+$CD38$^+$, median (IQR)**	11 (8, 21)	5 (3, 7)
**% CD8 HLADR$^+$CD38$^+$ (%)**	39 (26.25, 54.5)	13 (9, 26)
**% (total cells) CD45$^-$CD34$^+$**	0.059 (0.018, 0.21)	0.039 (0.0044, 0.091)
**% (total cells) CD45^{dim}CD34$^+$**	0.069 (0.011, 0.15)	0.087 (0.048, 0.178)

* n= 7 participants were on cART

# demographics data, activation markers (HLADR, CD38) and cART was not available in 1 out 19 participants

Table S2. Flow cytometry panels

Specificity	Fluorochrome	Clone	Cat. #	Manufacturer
**Panel Cohort Table 1**				
LIN-4*	FITC		562722	BD Biosciences
CD31	BUV395	WM59	565290	BD Biosciences
CD45	BUV805	HI30	564914	BD Biosciences
CD34	BB700	581	745835	BD Biosciences
CXCR4	BV421	12G5	306518	Biolegend
CD105	BV510	266	747746	BD Biosciences
CD49f	BV605	GoH3	740416	BD Biosciences
CD133	BV650	293C3	747567	BD Biosciences
CD146	BV711	P1H12	563186	BD Biosciences
PDL-1	BV786	MIH1	563739	BD Biosciences
CD14	APC	M5E2	301808	Biolegend
CX3CR1	APC-CY7	2A9-1	341615	Biolegend
CD309	PE	89106	FAB357P	R&D
CD202b	PE-vio770	REA198	130-101-614	Miltenyi
CD8	BUV496	RPA-TA	564804	BD Biosciences
CD3	BUV737	UCHT1	612750	BD Biosciences
**Specificity**	**Fluorochrome**	**Clone**	**Cat. #**	**Manufacturer**
**Panel Cohort Table S1**				
LIN-4*	FITC		562722	BD Biosciences
CD31	BUV395	WM59	565290	BD Biosciences
CD45	BUV805	HI30	564914	BD Biosciences
CD34	BB700	581	745835	BD Biosciences
CXCR4	APC	12G5	560936	BD Biosciences
CD105	BV510	266	747746	BD Biosciences
CD49f	BV605	GoH3	740416	BD Biosciences
CD133	BV421	293C3	566595	BD Biosciences
CD146	BV711	P1H12	563186	BD Biosciences
PDL-1	BV786	MIH1	563739	BD Biosciences
CX3CR1	APC-CY7	2A9-1	341615	Biolegend
CD309	PE/dazzle 594	7D4-6	359917	Biolegend
CD202b	PE-vio770	REA198	130-101-614	Miltenyi
CD117	BUV496	YB5.B8	565195	BD Biosciences

***LIN4 cocktail**: CD2, CD3, CD4, CD7, CD8, CD10, CD11b, CD14, CD19, CD20, CD56 and CD235a)

Table S3. Association between serum biomarkers of inflammation and circulating LIN4-CD45$^-$CD34$^+$ and LIN4$^-$CD45^{dim}CD34$^+$

Biomarker	Subset population (% of Total Cells)	*P*-value	Pearson-coefficient	95% CI for the Pearson-coefficient
IL-8 (pg/mL)	LIN4$^-$CD45$^-$CD34$^+$	0.404	0.143	-0.194 to 0.450
IL-8 (pg/mL)	LIN4$^-$CD45^{dim}CD34$^+$	0.417	-0.140	-0.447 to 0.198
hsIL-6 (pg/mL)	LIN4$^-$CD45$^-$CD34$^+$	0.473	-0.123	-0.434 to 0.213
hsIL-6 (pg/mL)	LIN4$^-$CD45^{dim}CD34$^+$	0.174	-0.232	-0.520 to 0.104
IL-6R (ng/mL)	LIN4$^-$CD45$^-$CD34$^+$	0.456	0.128	-0.209 to 0.438
IL-6R (ng/mL)	LIN4$^-$CD45^{dim}CD34$^+$	0.134	0.255	-0.080 to 0.538
TNFα (pg/mL)	LIN4$^-$CD45$^-$CD34$^+$	0.695	-0.068	-0.387 to 0.266
TNFα (pg/mL)	LIN4$^-$CD45^{dim}CD34$^+$	0.374	0.153	-0.185 to 0.458
TNFRI (ng/mL)	LIN4$^-$CD45$^-$CD34$^+$	0.461	-0.127	-0.137 to 0.496
TNFRI (ng/mL)	LIN4$^-$CD45^{dim}CD34$^+$	0.437	-0.134	-0.414 to 0.236
hsCRP (mg/mL)	LIN4$^-$CD45$^-$CD34$^+$	0.738	0.058	-0.276 to 0.379
hsCRP (mg/mL)	LIN4$^-$CD45^{dim}CD34$^+$	0.578	0.096	-0.240 to 0.411
sCD163 (mg/mL)	LIN4$^-$CD45$^-$CD34$^+$	0.603	-0.090	-0.406 to 0.246
sCD163 (mg/mL)	LIN4$^-$CD45^{dim}CD34$^+$	0.781	0.048	-0.285 to 0.370
sCD14 (mg/mL)	LIN4$^-$CD45$^-$CD34$^+$	0.043	-0.339	-0.600 to -0.011
sCD14 (mg/mL)	LIN4$^-$CD45^{dim}CD34$^+$	0.483	-0.121	-0.432 to 0.216
D-dimer (ng/mL)	LIN4$^-$CD45$^-$CD34$^+$	0.518	0.111	-0.225 to 0.424
D-dimer (ng/mL)	LIN4$^-$CD45^{dim}CD34$^+$	0.232	-0.204	-0.499 to 0.133
TFPI (ng/mL)	LIN4$^-$CD45$^-$CD34$^+$	0.821	0.039	-0.293 to 0.363
TFPI (ng/mL)	LIN4$^-$CD45^{dim}CD34$^+$	0.504	0.115	-0.221 to 0.427
sICAM1 (μg/mL)	LIN4$^-$CD45$^-$CD34$^+$	0.398	-0.145	-0.452 to 0.192
sICAM1(μg/mL)	LIN4$^-$CD45^{dim}CD34$^+$	0.284	0.183	-0.154 to 0.482
sVCAM1 (μg/mL)	LIN4$^-$CD45$^-$CD34$^+$	0.179	-0.229	-0.518 to 0.107
sVCAM1 (μg/mL)	LIN4$^-$CD45^{dim}CD34$^+$	0.129	0.258	-0.077 to 0.540

Abbreviations: IL-8: interleukin 8; IL-6: interleukin 6; IL-6R: interleukin 6 Receptor; TNF: Tumor Necrosis Factor; TNFRI: Tumor Necrosis Factor Receptor I, hsCRP: high sensitivity C-Reactive Protein; sCD163: soluble CD163; sCD14: soluble CD14; TFPI: Tissue Factor Pathway Inhibitor; sICAM1: soluble intercellular adhesion molecule 1; sVMAC1: soluble vascular adhesion molecule 1. *P* value <0.01 was considered significant.

Table S4. Association between lipids and circulating LIN4$^-$CD45$^-$CD34$^+$ and LIN4$^-$CD45$^+$CD34$^+$

Marker	Subset population (% of Total Cells)	*P*-value	Pearson-coefficient	95% CI for the Pearson-coefficient
Age	LIN4$^-$CD45$^-$CD34$^+$	0.817	0.040	-0.292 to 0.363
Age	LIN4$^-$CD45^{dim}CD34$^+$	0.895	-0.023	-0.348 to 0.308
Smoke	LIN4$^-$CD45$^-$CD34$^+$	0.271	-0.189	-0.486 to 0.149
Smoke	LIN4$^-$CD45^{dim}CD34$^+$	0.539	-0.106	-0.419 to 0.230
SBP	LIN4$^-$CD45$^-$CD34$^+$	0.315	0.172	-0.165 to 0.474
SBP	LIN4$^-$CD45^{dim}CD34$^+$	0.319	-0.171	-0.472 to 0.167
DM	LIN4$^-$CD45$^-$CD34$^+$	0.004	0.473	0.170 to 0.693
DM	LIN4$^-$CD45^{dim}CD34$^+$	0.367	-0.155	-0.460 to 0.182
BMI (Kg/m^2)	LIN4$^-$CD45$^-$CD34$^+$	0.965	-0.008	-0.335 to 0.321
BMI	LIN4$^-$CD45^{dim}CD34$^+$	0.713	0.064	-0.270 to 0.384
FRS	LIN4$^-$CD45$^-$CD34$^+$	0.835	-0.036	-0.360 to 0.296
FRS	LIN4$^-$CD45^{dim}CD34$^+$	0.923	-0.017	-0.343 to 0.313
Cholesterol (mg/dL)	LIN4$^-$CD45$^-$CD34$^+$	0.311	-0.174	-0.475 to 0.164
Cholesterol (mg/dL)	LIN4$^-$CD45^{dim}CD34$^+$	0.001	0.530	0.243 to 0.731
LDL (mg/dL)	LIN4$^-$CD45$^-$CD34$^+$	0.262	-0.192	-0.489 to 0.145
LDL (mg/dL)	LIN4$^-$CD45^{dim}CD34$^+$	0.000	0.645	0.401 to 0.803
HDL (mg/dL)	LIN4$^-$CD45$^-$CD34$^+$	0.964	0.008	-0.321 to 0.335
HDL (mg/dL)	LIN4$^-$CD45^{dim}CD34$^+$	0.251	-0.196	-0.493 to 0.141
Triglycerides (mg/dL)	LIN4$^-$CD45$^-$CD34$^+$	0.705	-0.065	-0.385 to 0.268
Triglycerides (mg/dL)	LIN4$^-$CD45^{dim}CD34$^+$	0.004	0.466	0.161 to 0.68

Abbreviations: SBP: systolic blood pressure; DM: Diabetes Mellitus, BMI: Body mass index, FRS Framingham Risk Score; LDL: Low-Density Lipoprotein cholesterol; HDL: High-Density Lipoprotein cholesterol. *P* value <0.01 was considered significant.

Table S5. Association between serum biomarkers of inflammation and LIN4-CD45^{-} CD34^{+} and LIN4^{-}CD45^{dim}CD34^{+} phenotypes

Biomearker	Expression (MFI) population	*P*-value	Pearson-coefficient	95% CI for the Pearson-coefficient
**LIN4^{-}CD45^{-}CD34^{+}**				
hsCRP (mg/mL)	CD309	0.002	0.582	0.251 to 0.790
hsCRP (mg/mL)	CD202b	0.101	0.342	-0.071 to 0.655
hsCRP (mg/mL)	CD31	0.346	0.169	-0.185 to 0.484
hsCRP (mg/mL)	CXCR4	0.034	0.374	0.029 to 0.639
hsCRP (mg/mL)	CD105	0.021	0.417	0.067 to 0.675
hsCRP (mg/mL)	CD49f	0.137	0.269	-0.088 to 0.564
hsCRP (mg/mL)	CD133	0.834	0.038	-0.309 to 0.376
hsCRP (mg/mL)	CD146	0.783	-0.057	-0.436 to 0.338
hsCRP (mg/mL)	PD-L1	0.004	0.507	0.180 to 0.733
hsIL-6 (pg/mL)	CD309	0.022	0.446	0.070 to 0.710
hsIL-6 (pg/mL)	CD202b	0.972	-0.008	-0.409 to 0.396
hsIL-6 (pg/mL)	CD31	0.744	-0.059	-0.394 to 0.290
hsIL-6 (pg/mL)	CXCR4	0.554	-0.109	-0.440 to 0.249
hsIL-6 (pg/mL)	CD105	0.009	0.465	0.125 to 0.706
hsIL-6 (pg/mL)	CD49f	0.804	-0.046	-0.388 to 0.308
hsIL-6 (pg/mL)	CD133	0.544	-0.109	-0.436 to 0.243
hsIL-6 (pg/mL)	CD146	0.779	0.057	-0.337 to 0.435
hsIL-6 (pg/mL)	PD-L1	0.719	-0.068	-0.418 to 0.299
**LIN4^{-}CD45^{dim}CD34^{+}**				
hsIL-6 (pg/mL)	CD309	0.498	-0.116	-0.428 to 0.220
hsIL-6 (pg/mL)	CD202b	0.800	-0.064	-0.515 to 0.415
hsIL-6 (pg/mL)	CD31	0.829	-0.037	-0.361 to 0.295
hsIL-6 (pg/mL)	CXCR4	0.228	0.206	-0.132 to 0.500
hsIL-6 (pg/mL)	CD105	0.033	0.362	0.033 to 0.620
hsIL-6 (pg/mL)	CD49f	0.155	0.242	-0.094 to 0.528
hsIL-6 (pg/mL)	CD133	0.593	0.092	-0.244 to 0.408
hsIL-6 (pg/mL)	CD146	0.058	0.339	-0.012 to 0.614
hsIL-6 (pg/mL)	PD-L1	0.005	0.459	0.155 to 0.684
TNFα (pg/mL)	CD309	0.084	0.291	-0.0405938 to
TNFα (pg/mL)	CD202b	0.440	0.194	-0.2999285 to
TNFα (pg/mL)	CD31	0.072	-0.303	-0.574 to 0.028
TNFα (pg/mL)	CXCR4	0.912	-0.311	-0.311 to 0.345
TNFα (pg/mL)	CD105	0.136	-0.084	-0.083 to 0.543
TNFα (pg/mL)	CD49f	0.815	-0.364	-0.363 to 0.292
TNFα (pg/mL)	CD133	0.868	-0.354	-0.353 to 0.303
TNFα (pg/mL)	CD146	0.242	-0.147	-0.146 to 0.523
TNFα (pg/mL)	PD-L1	0.003	0.177	0.177 to 0.697

TNFRI (ng/mL)	CD309	0.624	-0.084	-0.402 to 0.250
TNFRI (ng/mL)	CD202b	0.927	-0.023	-0.485 to 0.448
TNFRI (ng/mL)	CD31	0.812	-0.041	-0.365 to 0.291
TNFRI (ng/mL)	CXCR4	0.055	0.322	-0.007 to 0.588
TNFRI (ng/mL)	CD105	0.253	0.198	-0.144 to 0.498
TNFRI (ng/mL)	CD49f	0.862	0.029	-0.301 to 0.355
TNFRI (ng/mL)	CD133	0.790	0.045	-0.287 to 0.368
TNFRI (ng/mL)	CD146	0.195	0.234	-0.123 to 0.539
TNFRI (ng/mL)	PD-L1	0.005	0.454	0.1472 to 0.681

Abbreviations: hsIL-6: interleukin 6; TNFa: Tumor Necrosis Factor Alpha; TNFRI: Tumor Necrosis Factor Receptor I, hsCRP: high sensitivity C-Reactive Protein. *P* value <0.01 was considered significant

Table S6. Angiogenic CD4 and CD8 T cells

Biomarker	Expression (MFI) population	*P*-value	Pearson-coefficient	95% CI for the Pearson-coefficient
FRS 10 yr %	$CD4T_{angs}$	0.045	-0.346	-0.612, -0.008
FRS 10 yr %	$CD8T_{angs}$	0.044	-0.347	-0.613, -0.009
SBP (mmHg)	$CD4T_{angs}$	0.001	-0.510	-0.718, -0.218
SBP (mmHg)	$CD8T_{angs}$	0.023	-0.377	-0.628, -0.056
DBP (mmHg)	$CD4T_{angs}$	0.014	-0.407	-0.649, -0.091
DBP (mmHg)	$CD8T_{angs}$	0.018	-0.391	-0.638, -0.072
D-dimer mg/L	$CD4T_{angs}$	0.015	-0.401	-0.644, 0.084
D-dimer mg/L	$CD8T_{angs}$	0.579	-0.095	-0.411, 0.240

Abbreviations: FRS: Framingham Risk Score; SBP: Systolic Blood Pressure; DBP: Diastolic Blood Pressure. *P* value <0.01 was considered significant

Supplementary Figure Legends

Figure S1. Detection of circulating cell progenitors in fresh and frozen PBMCs

Fresh and frozen PBMCs from the same healthy volunteers (n= 6) were stained with LIVE/DEAD followed by a cocktail of mAbs: LIN4 (LIN4 cocktail: CD2, CD3, CD4, CD7, CD8, CD10, CD11b, CD14, CD19, CD20, CD56, CD235a), CD45 and CD34. Full minus one (FMO) was used for gating CD34 and surface markers. **(A)** Representative gating strategy. **(B)** Representative dot plots and gating strategy of LIN4$^-$, LIN4$^-$CD45$^-$ and LIN4$^-$CD45$^+$ and **(C)** FMO control and CD34 staining. Frequencies of: **(D)** LIN4$^-$ cells, **(E)** LIN4$^-$CD45$^-$ and LIN4$^-$CD45$^+$, **(F)** LIN4$^-$CD45$^-$CD34$^+$ cells and LIN4$^-$CD45^{dim}CD34$^+$. Proportions are expressed as frequency of total live cells. Whiskers represent median and IQR. Comparison between groups was performed using nonparametric Mann-Whitney test. *P* value < 0.05 was considered significant.

Figure S2. Constant Proportion of Circulating Endothelial Progenitors in PBMCs from PWH.

PBMCs from HIV infected patients (n= 19) at two time points separated by six months (T1 and T2). PBMCs were thawed and rested overnight and stained with LIVE/DEAD followed by a cocktail of mAbs: LIN4, CD45, CD34. Full minus one (FMO) was used for gating CD34 as described Figure S1. **(A)** Percentage of the cell subsets LIN4$^-$, LIN4$^-$CD45$^-$ and LIN4$^-$CD45$^+$ expressed as frequency of total live cells in both time points (T1 and T2). **(B)** Percentage of LIN4$^-$CD45$^-$CD34$^+$ cells and LIN4$^-$CD45^{dim}CD34$^+$ expressed as frequency of total live cells. Comparison between Time point 1 (T1) and time point 2 (T2) was performed using paired non-parametric Wilcoxon test. *P* value < 0.05 was considered significant.

Figure S3. Phenotype of $LIN4^-CD45^-CD34^+$ cells and $LIN4^-CD45^{dim}CD34^+$ Circulating progenitor cells from PWH.

PBMCs from PWH (n= 36) were thawed and rested overnight. PBMCS were stained with LIVE/DEAD followed by a cocktail of mAbs described in Table Flow Cytometry Panel including: LIN4, CD45, CD3, CD8, CD34, CD31,CD105, CD49f, CD133, CD146, PDL-1, CD14, CD309 and CD202b (Table S1). Full minus one (FMO) was used control. **(A)** Representative example of the gating strategy of FMOs and positive staining examples of the frequencies and median fluorescence intensity (MFI) of $LIN4^-CD45^-CD34^+$ (Left panel) and $LIN4^-CD45^{dim}CD34^+$ (Right panel). **(B)** Expression of the markers CD31, CXCR4, CD49f, CD309, CD202b, CX3CR1, CD105, CD133, CD146 and PD-L1 in the $LIN4^-CD45^-CD34^+$ (closed red circle) cells and $LIN4^-CD45^{dim}CD34^+$ (open red circle) are shown as frequency of the parent. Comparison between groups was performed using nonparametric Wilcoxon test. *P* value < 0.05 was considered significant.

Figure S4. Phenotype of circulating cell progenitors $LIN4^-CD45^-CD34^+$ and $LIN4^-CD45^{dim}CD34^+$ in PWH.

PBMCs from healthy control (HC, n=10), PWH with viral loads < 50 copies/ml (n= 12), and > 50 copies/ml (n= 19) were thawed and rested overnight. PBMCs were stained with LIVE/DEAD followed by a cocktail of mAbs: LIN4, CD45, CD34, CD31, CXCR4, CD105, CD49f, CD133, CD146, PDL-1, CD309, CD117 and CD202b. Surface expression of: **(A)** $LIN4^-CD45^-CD34^+$ and **(B)** $LIN4^-CD45^{dim}CD34^+$ cells. Expression of the markers are shown as frequency of the parent population. Whiskers represent median and IQR. Comparison between groups was performed using non-parametric Mann Whitney test. *P* value < 0.05 was considered significant.

Figure S1

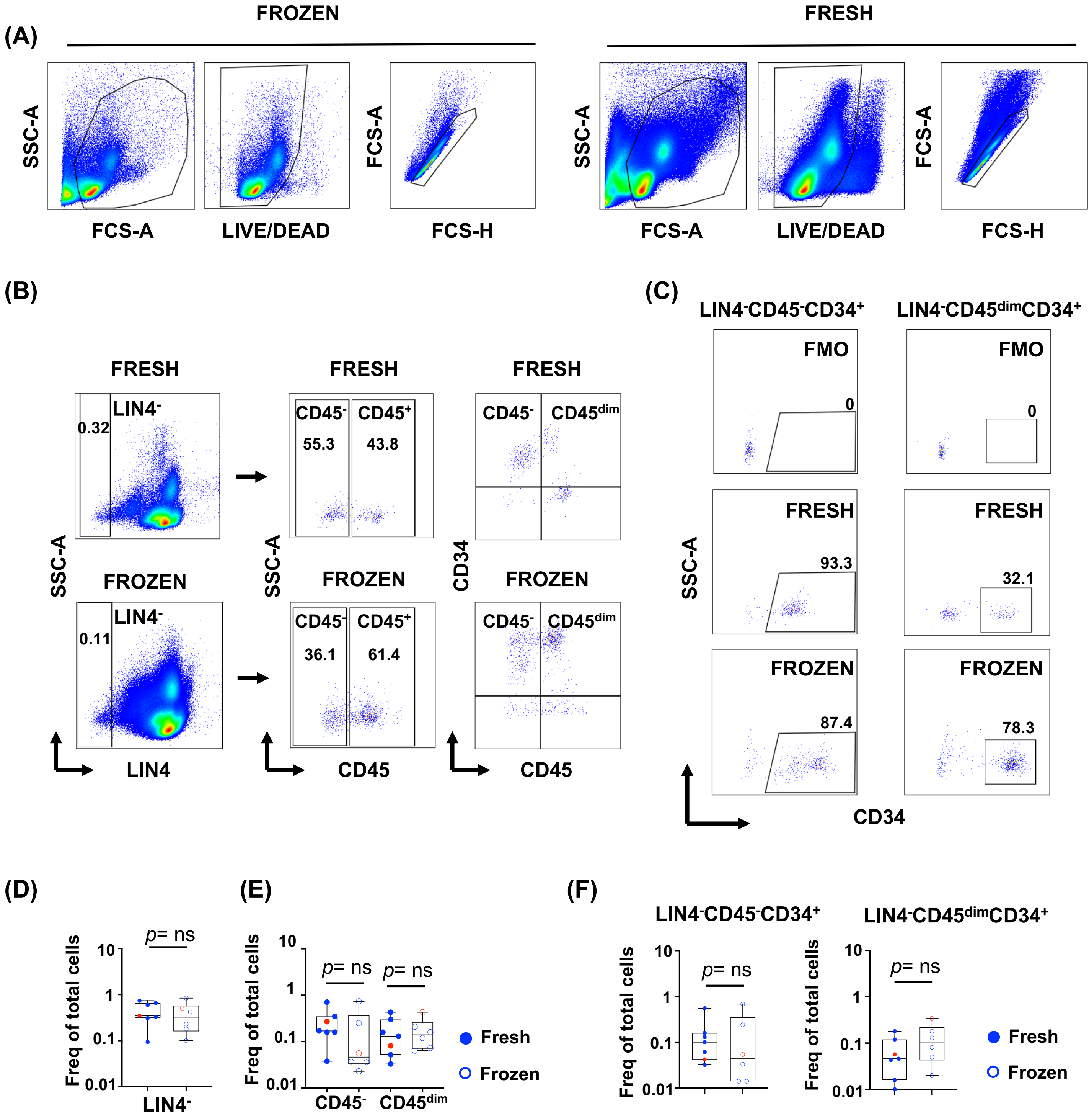
(A)
FROZEN
FRESH
SSC-A
FCS-A
LIVE/DEAD
FCS-A
FCS-H
(B)
FRESH
LIN4-
0.32
CD45- CD45+
55.3 43.8
CD45- CD45dim
FROZEN
LIN4-
0.11
36.1 61.4
SSC-A
CD34
LIN4
CD45
(C)
LIN4-CD45-CD34+
LIN4-CD45dimCD34+
FMO
0
93.3
32.1
87.4
78.3
CD34
(D)
Freq of total cells
p= ns
10
1
0.1
0.01
LIN4-
(E)
CD45- CD45dim
Fresh
Frozen
(F)
LIN4-CD45-CD34+
LIN4-CD45dimCD34+

Figure S2

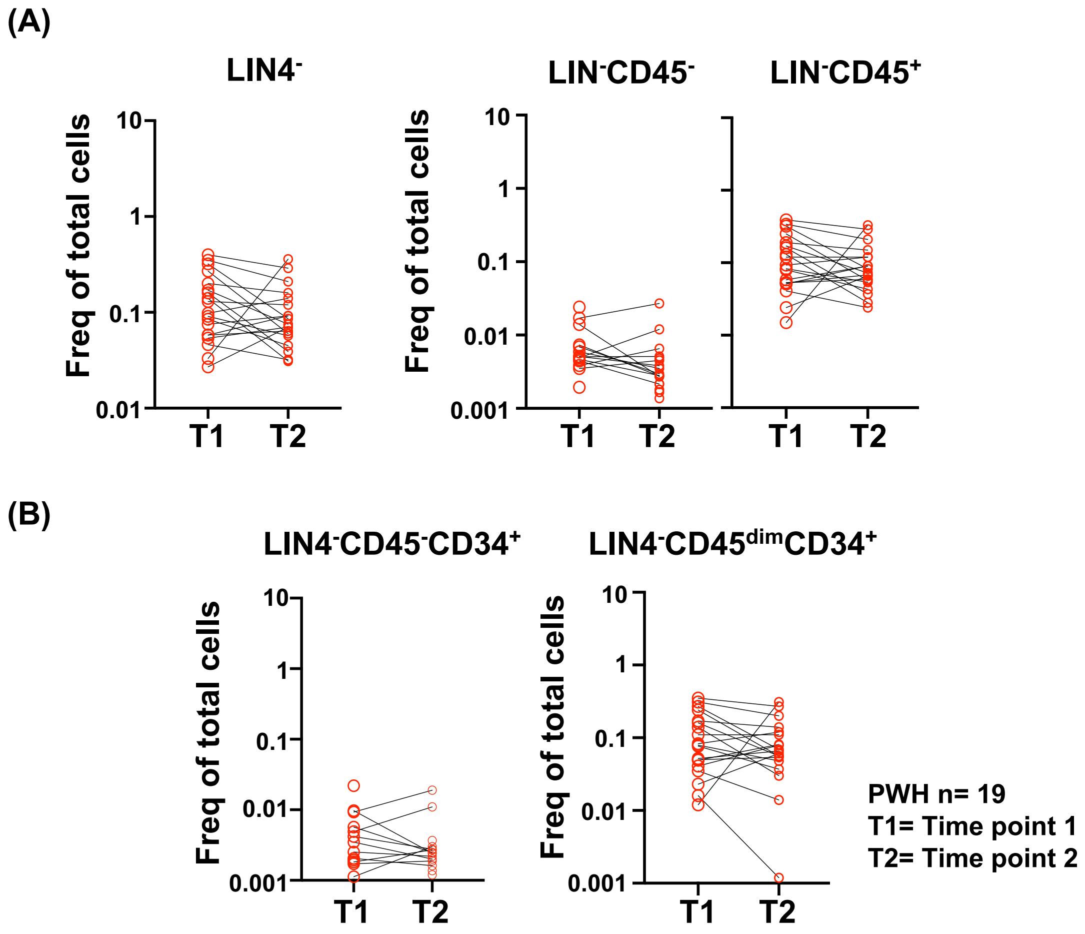
(A)
LIN4⁻
LIN⁻CD45⁻
LIN⁻CD45⁺
Freq of total cells
10
1
0.1
0.01
0.001
T1
T2
(B)
LIN4⁻CD45⁻CD34⁺
LIN4⁻CD45^dim CD34⁺
PWH n= 19
T1= Time point 1
T2= Time point 2

Figure S3

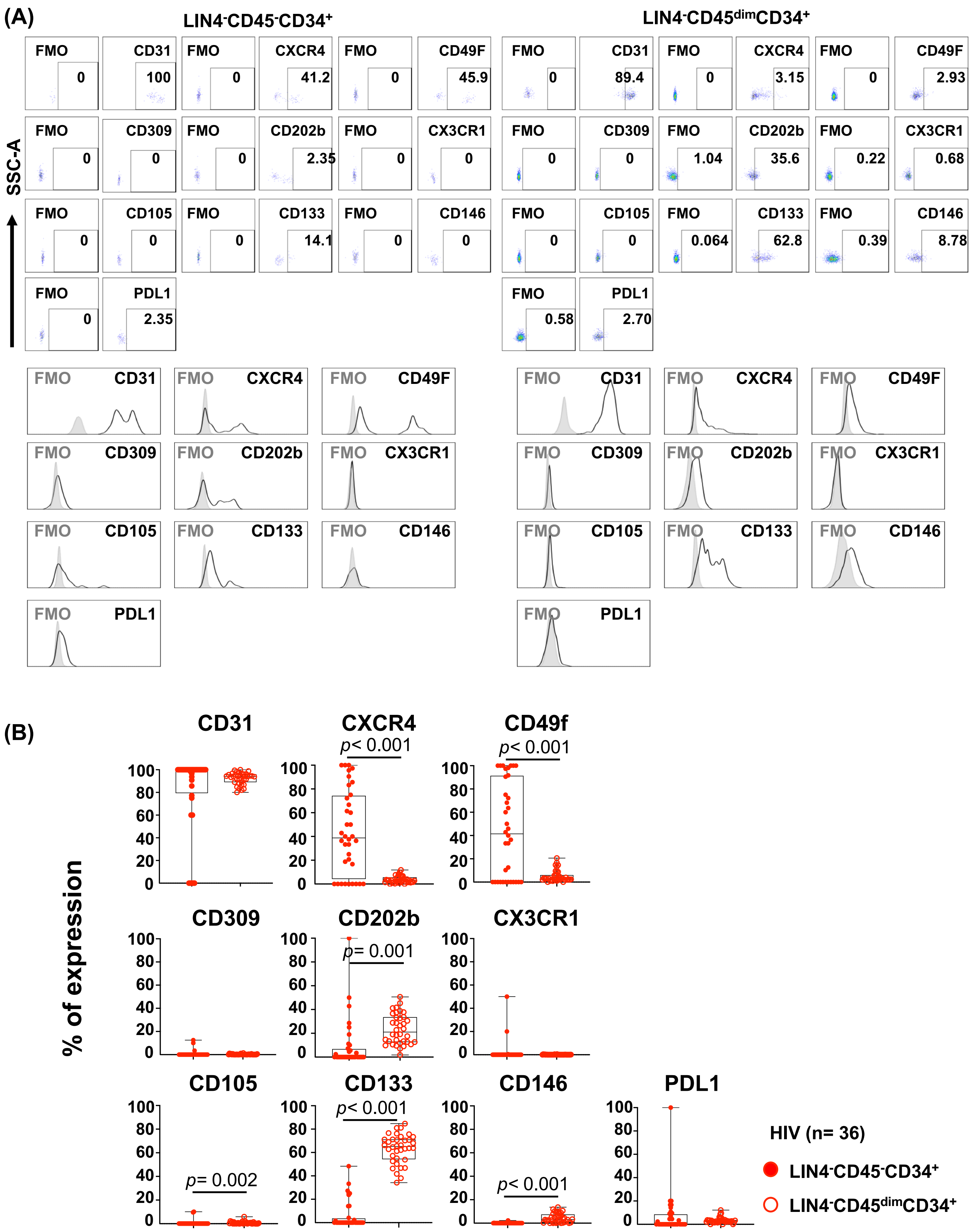
(A)
LIN4-CD45-CD34+
LIN4-CD45dimCD34+
SSC-A
FMO 0
CD31 100
FMO 0
CXCR4 41.2
FMO 0
CD49F 45.9
FMO 0
CD31 89.4
FMO 0
CXCR4 3.15
FMO 0
CD49F 2.93
FMO 0
CD309 0
FMO 0
CD202b 2.35
FMO 0
CX3CR1 0
FMO 0
CD309 0
FMO 1.04
CD202b 35.6
FMO 0.22
CX3CR1 0.68
FMO 0
CD105 0
FMO 0
CD133 14.1
FMO 0
CD146 0
FMO 0
CD105 0
FMO 0.064
CD133 62.8
FMO 0.39
CD146 8.78
FMO 0
PDL1 2.35
FMO 0.58
PDL1 2.70
FMO CD31
FMO CXCR4
FMO CD49F
FMO CD309
FMO CD202b
FMO CX3CR1
FMO CD105
FMO CD133
FMO CD146
FMO PDL1
(B)
% of expression
CD31
CXCR4 p< 0.001
CD49f p< 0.001
CD309
CD202b p= 0.001
CX3CR1
CD105 p= 0.002
CD133 p< 0.001
CD146 p< 0.001
PDL1
0 20 40 60 80 100
HIV (n= 36)
LIN4-CD45-CD34+
LIN4-CD45dimCD34+

Figure S4

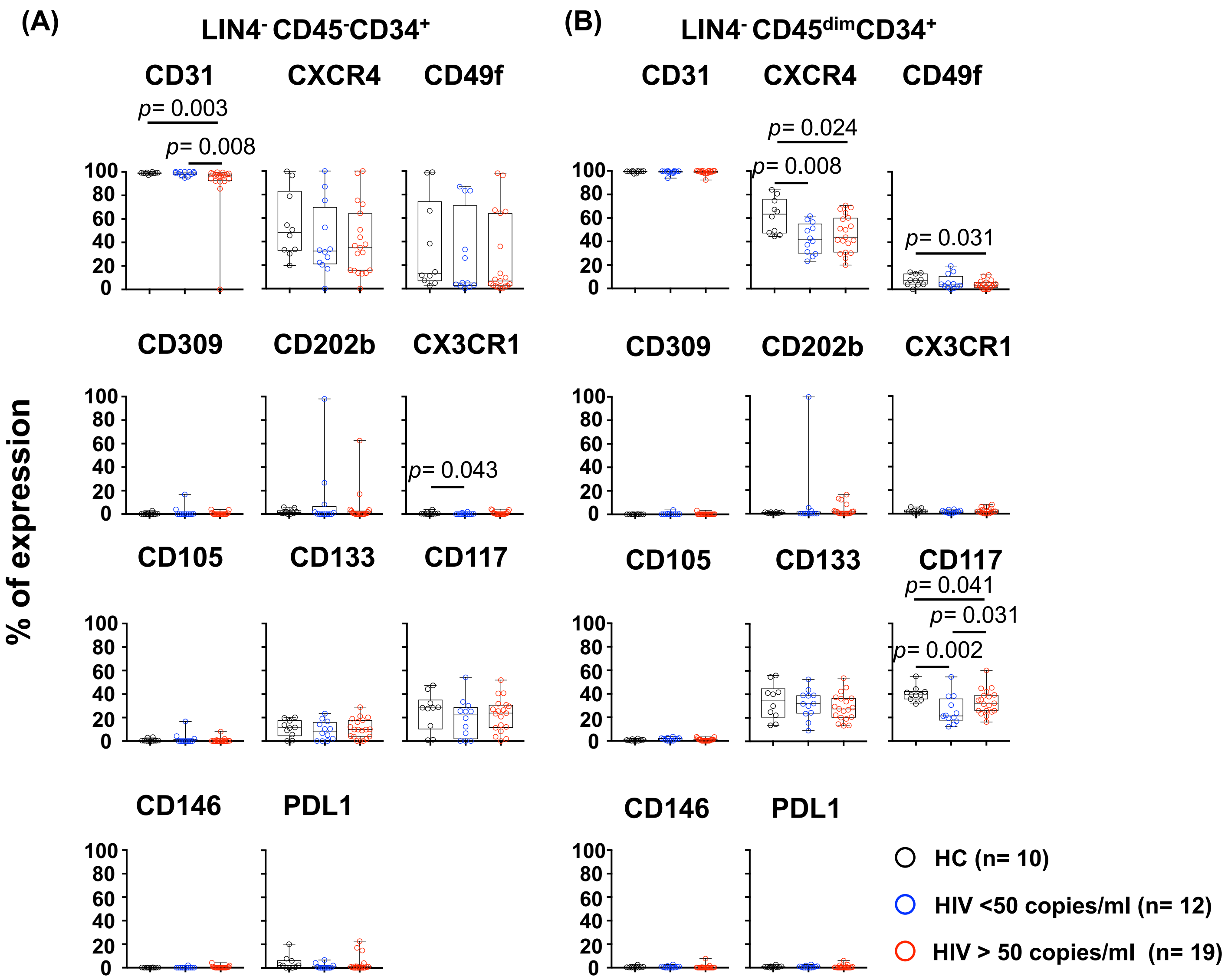
(A)
LIN4- CD45-CD34+
(B)
LIN4- CD45dimCD34+
CD31
CXCR4
CD49f
CD309
CD202b
CX3CR1
CD105
CD133
CD117
CD146
PDL1
p= 0.003
p= 0.008
p= 0.043
p= 0.024
p= 0.008
p= 0.031
p= 0.041
p= 0.031
p= 0.002
% of expression
HC (n= 10)
HIV <50 copies/ml (n= 12)
HIV > 50 copies/ml (n= 19)
